# A severe COVID-19 case with schizophrenia as well as other chronic diseases

**DOI:** 10.1590/1414-431X202010426

**Published:** 2021-01-15

**Authors:** Lizhu Zeng, Huagen Zhang, Yongjun He, Bifa Lai, Zhen Huang, Li Lin, Zhixiong Zhong, Xuemin Guo

**Affiliations:** 1 Meizhou People's Hospital, Meizhou China Meizhou People's Hospital, Meizhou, China; 2 Guangdong Provincial Key Laboratory of Precision Medicine and Clinical Translational Research of Hakka Population, Meizhou China Guangdong Provincial Key Laboratory of Precision Medicine and Clinical Translational Research of Hakka Population, Meizhou, China

**Keywords:** Severe COVID-19, Schizophrenia, Lymphocyte subset, Serological assay

## Abstract

The prognosis of COVID-19 (coronavirus disease 2019) is usually poor when it occurs in aged adults or in patients with chronic diseases, which brought a great challenge to clinical practice. Furthermore, widespread depression, anxiety, and panic related to SARS-CoV-2 (severe acute respiratory syndrome-coronavirus 2) infection affected treatment compliance and recovery. Here we report the successful treatment of a 57-year-old male with severe COVID-19, schizophrenia, hypertension, and type 2 diabetes. The patient's negative emotions (such as tension, panic, and anxiety), particularly his aggression and paranoia, seriously hindered treatment, leading to a deteriorating condition. Psychological counseling and supportive psychotherapy were given but the effect was weak. To improve adherence, risperidone and quetiapine fumarate were replaced by olanzapine for anti-schizophrenic treatment to reduce insomnia and anxiety side effects, associated with sedative-hypnotic drugs as well as psychological counseling. The treatment compliance of the patient improved significantly. The patient's serum alanine aminotransferase increased abnormally in the late stage of hospitalization, suggesting potential liver damage after complex medication strategies. We also monitored the changes of lymphocyte subsets and retrospectively analyzed the virus-specific antibody response. The results suggested that dynamic monitoring of lymphocyte subsets and virus-specific antibody response could facilitate disease progression evaluation and timely treatment plan adjustments. An effective psychotropic drug intervention associated with psychological counselling and psychotherapy are essential for the successful adherence, treatment, and rehabilitation of psychiatric disorders in COVID-19 patients.

## Introduction

With the fast spreading of the severe acute respiratory syndrome coronavirus 2 (SARS-CoV-2) virus, coronavirus disease 2019 (COVID-19) has become a global pandemic. As of August 30, 2020, the number of laboratory-confirmed cases exceeded twenty-five million (1). About 20% COVID-19 patients have severe or critical disease, with a rough mortality rate up to 3.36% (1,2). Severe and critical COVID-19 is prone to occur in people with low immunity or chronic diseases, which brought a great challenge to clinical practice (2,3). Meanwhile, widespread depression, anxiety, and panic related to SARS-CoV-2 infection also hindered treatment compliance and recovery (4–6).

Here, we present the disease progression and treatment of a severe COVID-19 patient with schizophrenia, type 2 diabetes, and hypertension. The change in peripheral blood lymphocyte subsets was monitored while hospitalized, and the virus-specific immunoglobulin M (IgM) and IgG response was retrospectively analyzed.

## Case presentation

### History and examination

A Chinese male in his late 50s developed a fever, with a mild cough, shortness of breath, and chills on January 25, 2020. Due to persistent fever, he was admitted to our hospital on February 1, 2020. Medical records showed that the patient had hypertension (taking metoprolol and nifedipine tablets daily), type 2 diabetes (taking metformin and glyburide tablets daily), and schizophrenia (taking risperidone and quetiapine fumarate tablets daily). He reported that the control of these chronic diseases was acceptable, but his mental state was poor, mainly manifested as depression, anxiety, and insomnia. Physical examination showed slight overweight (80 kg), body mass index (BMI) of 28.34, body temperature of 37°C, heart rate of 80 bpm, breathing rate of 20 times/min, blood pressure of 120/68 mmHg, poor appetite and sleep, and obvious schizophrenic symptoms. Chest computed tomography (CT) scan showed multiple ground-glass opacities in both lungs. Laboratory tests showed slightly high fibrinogen (FIB, 4.48 g/L, normal range 2.00-4.00 g/L) and slightly low arterial oxygen saturation (SaO_2_%, 93.1%, normal range 95.0-98.0%), arterial partial pressure of carbon dioxide (PaCO_2_, 33.9 mmHg, normal range 34.90-44.90 mmHg), and arterial partial pressure of oxygen (PaO_2_, 78.7 mmHg, normal range 83.20-108.00 mmHg). The patient was diagnosed as moderate COVID-19 after SARS-CoV-2 RNA was detected in his throat swab specimen.

### Treatment and complications

On admission, the patient was given oxygen therapy by nasal catheter to increase oxygen saturation, methylprednisolone to attenuate lung inflammation, and supportive psychotherapy. Meanwhile, the patient was treated with antivirals (lopinavir and ritonavir tablets), anti-schizophrenic drugs (quetiapine fumarate and risperidone tablets), antihypertensive agents (metoprolol and nifedipine SR tablets), antidiabetic agents (metformin sustained release tablets and insulin injection), and immunomodulators (IVIG and thymalfasin injection) (Supplementary Table S1). Due to the patient's severe depression, anxiety, panic, especially physical aggression, paranoia, and schizophrenic symptoms, the above symptomatic and supportive therapies were implemented arduously.

On the evening of February 3, i.e., 2 days after hospital admission, the patient's condition deteriorated, with shortness of breath (about 23 times/min) under nasal oxygen inhalation and low finger SPO_2_ (about 92%, normal value 95-100%). He was transferred to a negative pressure ward, following non-invasive ventilation rescue with ECG monitoring. Meanwhile, multiple complications occurred, including sequelae of cerebral infarction, type II respiratory failure, acute respiratory distress syndrome, hyperlactic acidemia, and electrolyte metabolism disorders. Chest CT showed extensive ground-glass opacities in both lungs. The intial moderate case developed into a severe COVID-19. To improve the treatment compliance of the case, quetiapine fumarate and risperidone were replaced by olanzapine to reduce anxiety, insomnia, and headache side effects (7,8); midazolam, diazepam, and dexmedetomidine were given to ease anxiety and help sleep. Additionally, supportive psychotherapy was strengthened. Arbidol and fluconazole were given to strengthen antiviral therapy. On February 6, the patient's clinical symptoms improved significantly, and improved further on February 9, except for occasional cough. During this period, human albumin injection was given to improve the low albumin condition, supplemented with furosemide to help the absorption of pulmonary interstitial effusion; low molecular weight heparin calcium injection was given to prevent venous thrombosis due to a high level of fibrinogen. On February 19, the patient was discharged after negative SARS-CoV-2 RNA results in the throat swab and the stool specimens for two consecutive days as well as improved inflammatory lesions in both lungs revealed by CT.

### Daily test of liver and kidney function

The patient received a complex medication scheme to cope with underlying diseases, virus infection, and condition deterioration during hospitalization. To monitor the potential side effects of the drugs, the main indicators of liver function and kidney function were detected. The results showed that the main indicators of renal function, including blood urea nitrogen and creatinine, remained within normal ranges, while alanine aminotransferase, as a main indicator of liver function, was normal in the early-middle stage of hospitalization but increased abnormally in the late stage (from day 19 of onset and day 12 after hospital admission) (Supplementary Table S1), suggesting potential liver damage due to complex medications.

### Dynamically monitoring lymphocyte subset change

**Figure 1:**
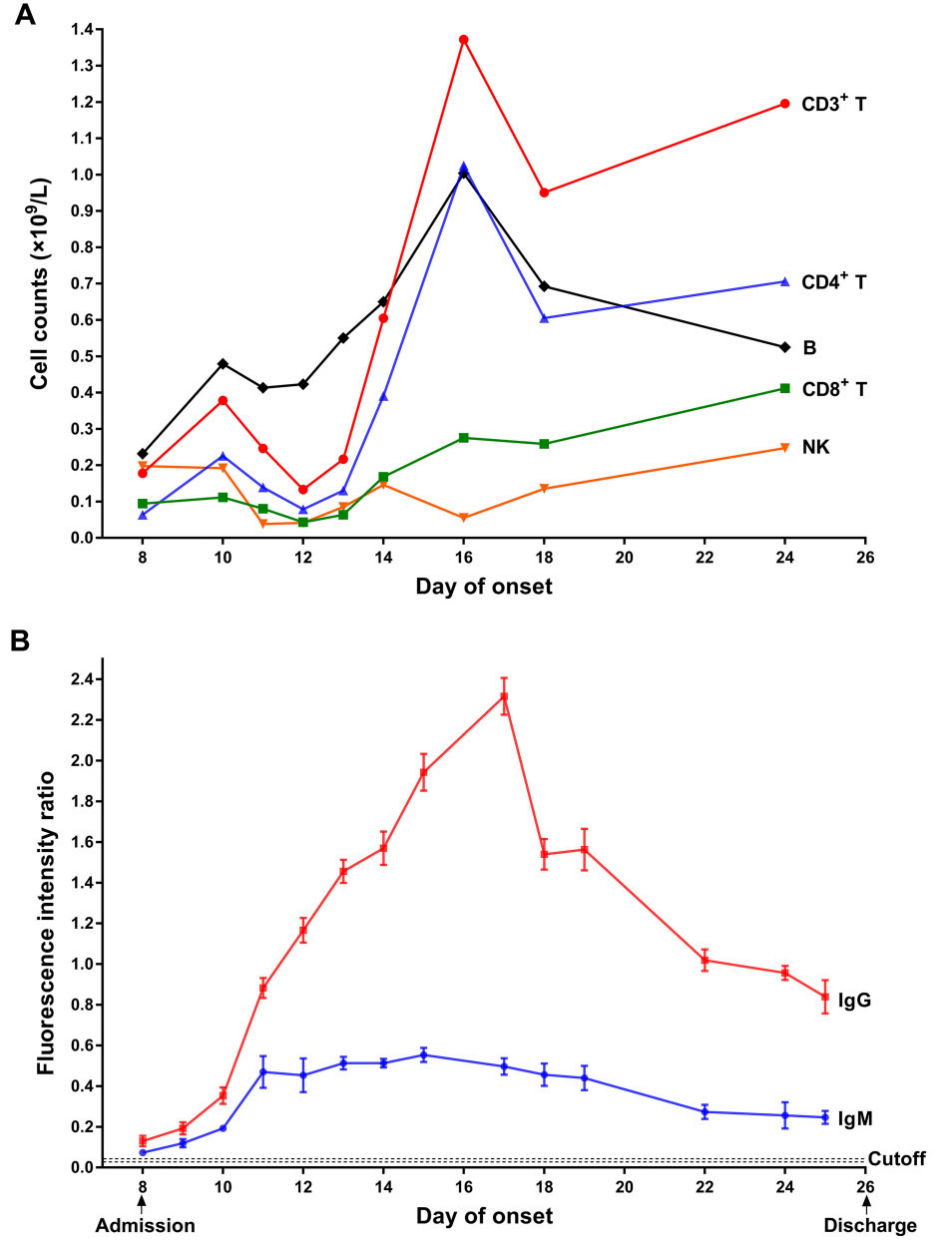
Dynamic monitoring the lymphocyte change and virus-specific antibody response. **A**, Flow-cytometry analysis of lymphocyte subsets. **B**, Serological assay of severe acute respiratory syndrome coronavirus 2 (SARS-CoV-2)-specific IgG and IgM. The presence and relative concentration of IgM and IgG targeting SARS-CoV-2 were measured by a specific fluorescent immunochromatography detection kit. The cutoff values for IgG and IgM detection were 0.057 and 0.067, respectively. B: B lymphocyte cell; NK: natural killer cell.

The peripheral lymphocyte subset of the severe case during hospitalization was monitored using flow cytometry. As shown in Figure 1A, the number of CD3^+^ T, CD4^+^ T, and CD8^+^ T cells was below the normal range on admission (day 8 of onset) and dropped further from day 10 of onset to day 12 accompanying COVID-19 deterioration, then bounced back to normal values on day 14 with the improvement of his physical condition, finally reached a peak on day 16, followed by a drop to normal range again on day 18 and thereafter. The B cell number showed a similar change during hospitalization and was higher than the normal value from day 14 to day 18. The natural killer (NK) cell counts changed weakly, fluctuating above and below normal value.

### Serological assay of SARS-CoV-2-specific IgG and IgM

Serial serum samples of this severe case collected during hospitalization were retrospectively analyzed by immunofluorescence chromatography assay kit for SARS-CoV-2-specific IgM and IgG (Zhongshan Chuangyi, China). As shown in Figure 1B, both the IgM and IgG antibodies were detectable on admission day; then IgM and especially IgG increased significantly on day 10 accompanying disease deterioration and reached a peak on day 11 and day 17, with about 40- and 140-fold increase, respectively, compared to the antibody level on admission. After that, IgM decreased but stayed in a steady state, while IgG decreased constantly. On discharge day, the IgM and IgG levels were about 30 times and 20 times higher than on admission day, respectively.

## Discussion

Studies have shown that the global COVID-19 outbreak, the fast spread of SARS-CoV-2, and the high rate of severe disease and death have caused serious anxiety, depression, and panic among patients, healthcare workers, and the general public worldwide (4–6). These negative emotions undoubtedly weaken treatment adherence. Here, we reported a Chinese severe COVID-19 patient with schizophrenia, hypertension, and type 2 diabetes. The patient's depression, anxiety, and insomnia on admission were most likely due to risperidone and quetiapine fumarate side effects (7,8). After confirming infection with SARS-CoV-2, the patient suffered a dramatic mood deterioration in the early stage of his hospitalization, including severe depression, anxiety, panic, physical aggression, paranoia, and schizophrenic symptoms. These negative emotions, particularly his aggression and paranoia, seriously hindered treatment. Psychological counseling and supportive psychotherapy were given but the effect was weak. We speculated that the patient's schizophrenic symptoms, insomnia, and anxiety deteriorated his disease progression and were responsible, at least in part, for the change from moderate to severe COVID-19. Given the side effects of insomnia, anxiety, and headache, the above medications were replaced with olanzapine for anti-schizophrenic treatment (Supplementary Table S1), while sedative-hypnotic drugs as well as psychological counseling were used together for symptomatic treatment. Then, treatment compliance by the patient improved significantly. Therefore, the effective psychotropic drug intervention was essential for improving treatment adherence and subsequent successful treatment of a COVID-19 patient with psychiatric disorders; psychological counseling and psychotherapy were indispensable for the rehabilitation of this COVID-19 patient as well.

It has been shown that about one fourth of COVID-19 cases in China have comorbidities (diabetes, hypertension, cardiovascular, and cerebrovascular diseases) that are related to poor prognosis (9–11). These complicated COVID-19 patients have to be given more medications to control the development of complications. Thus, the risk of impairment of hepatic and renal functions increases. The severe COVID-19 patient reported herein took more than a dozen drugs every day, including antihypertensive drugs, antidiabetic drugs, anti-schizophrenic drugs, glucocorticoids, immunomodulators, and drugs with potential antiviral activity, during hospitalization. His serum ALT was normal in the early and middle stages but abnormally high in the late stage of the disease, suggesting liver damage, which was likely caused by complicated mediations. The main indicators of renal function remained within normal ranges (Supplementary Table S1). Therefore, under complex medication conditions, monitoring the chemical indicators of liver and kidney function is of great significance for timely detection of abnormal conditions and adjustment of treatment programs.

Flow cytometry analysis of the peripheral lymphocyte subsets of the COVID-19 patient showed that the change of his T lymphocyte subsets during hospitalization was basically consistent with his disease progression. When his condition worsened, the T cell number reduced significantly (Figure 1A), which is consistent with the current reports (12–14), further providing experimental evidence that the significant reduction in T cell number could act as a critical indicator of deterioration. We also found that the number of the B cells and the level of the SARS-CoV-2-specific IgM and IgG changed accordingly (Figure 1B). Among the 11 COVID-19 patients admitted to our hospital, this patient was the severest case, and his virus-specific IgG and IgM showed the highest peak concentration. This result is in accordance with the previous observation that severe patients had stronger ability to produce SARS-CoV-2 specific antibodies than mild patients (14,15), but the underlying mechanism remains elusive. Experiments should be carried out to investigate the contribution of large number of antibodies on the rehabilitation of severe COVID-19 patients.

### Conclusion

An effective psychotropic drug intervention associated with psychological counselling and psychotherapy were essential for the successful adherence, treatment, and rehabilitation of psychiatric disorders of this COVID-19 patient. For severe COVID-19 patients with multiple chronic diseases, complex medication strategies may cause liver damage. Monitoring the changes of lymphocyte subsets and virus-specific antibodies simultaneously can facilitate the evaluation of disease progression and the timely adjustment of the treatment plan.

## Supplementary Material

Click here to view [pdf].
